# Professor Yongjia Duan: a distinguished plant pathologist and agricultural educator

**DOI:** 10.1007/s13238-019-0616-1

**Published:** 2019-03-26

**Authors:** Fei Du, Wei-Ping Deng, Xia-Hong He, Hong Cai, You-Yong Zhu

**Affiliations:** 1 State Key Laboratory for Conservation and Utilization of Bio-Resources in Yunnan, Yunnan Agricultural University, Kunming, 650201 China; 2 Key Laboratory for Agro-biodiversity and Pest Control of Ministry of Education, Yunnan Agricultural University, Kunming, 650201 China

Professor Yongjia Duan (段永嘉) is a distinguished plant pathologist and agricultural educator in China. He has devoted his entire life in developing and sharing his knowledge and experiences in Plant Pathology.

**Figure 1:**
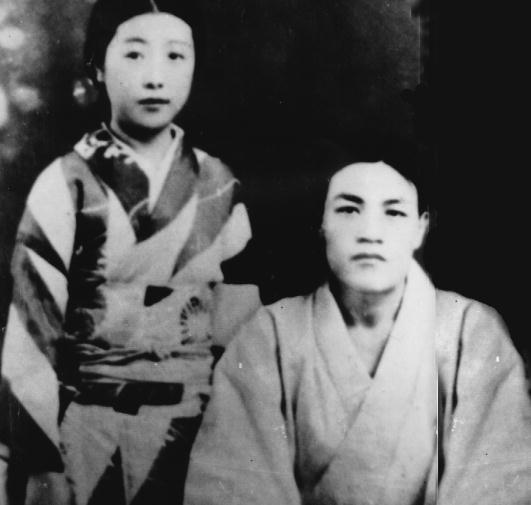
**Prof. Yongjia Duan and his wife**

Prof. Duan was born in 1910 in Siping, Jilin Province. In 1931, he went to Hokkaido University in Japan to study plant pathology, where he obtained his bachelor’s degree. In Japan, he met Ms. Hiroko Omura, a primary school teacher (Fig. 1). They fell in love and got married. In 1937, Prof. Duan and his wife went back to China, hoping that he could contribute to the development of science and technology in China.

Prof. Duan had teaching experience in several universities, such as Jiangsu Educational College, Guangxi University and Hunan Agricultural College. At the end of 1944, he was introduced to the Agricultural College at Yunnan University by Prof. Haiqiu Zhang (张海秋), the Dean of the Agricultural College. During that period, Prof. Duan developed deep friendships and scientific relationships with Profs. Fenlan Dai (戴芬澜) and Dafu Yu (俞大绂), the pioneers in the field of plant pathology; Prof. Shanbao Jin (金善宝), a famous wheat expert; Prof. Jueming Wang (汪厥明), a biostatistician; Prof. Wanjun Zheng (郑万钧), a forestry scientist and Prof. Xingyuan Lu (陆星垣), a sericulture expert. After the founding of the People’s Republic of China, Prof. Duan served as the head of the Agricultural College at Yunnan University. In 1958, the Agricultural College became independent of Yunnan University as Kunming Agriculture and Forestry College, and later on developed as Yunnan Agricultural University in 1970. In 1976, Prof. Duan became the head of the Department of Plant Protection at Yunnan Agricultural University. As the founder and lifelong contributor to higher agricultural education in Yunnan, even retiring in 1989, he never left education and research positions until he passed away at the age of 95.

**Figure 2:**
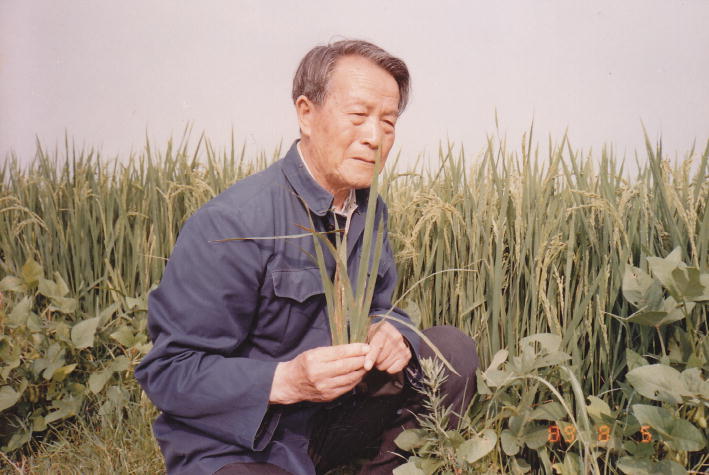
**Prof. Duan was working on collection and investigation of rice blast**

Prof. Duan is a well-known plant pathologist in China and the founder of the Department of Plant Pathology in Yunnan Province. In 1945, after the Second World War, lots of scientists and experts left Kunming, leading to a shortage of college teachers there. As the head of the Department of Agriculture, Prof. Duan actively looked for teachers to fulfill this shortage, and restored the development of higher education in Agriculture in Yunnan. He also started the course of plant pathology in the Department of Agriculture, which is now the plant pathology discipline of Yunnan Agricultural University. Due to the lack of teaching materials, he wrote and published “Phytopathogenic Bacteriology” in 1947, which was one of the earliest plant pathology textbook in China (Duan, 1947). He also encouraged the teachers and students to collect different disease specimens and set up places to preserve disease specimens (Fig. 2). Till now, some specimens are still well preserved. In 1957, a puzzling disease caused yellow leaves and growth retardation in rice in Xishuangbanna, Yunnan Province. After a thorough investigation, Prof. Duan identified the cause of the disease, provided prevention and control methods to the government and effectively controlled the spread of the diseases. In the same year, more than 30 kinds of crops and 70 pathogens were reported (Duan, 1957). In 1958, the first comprehensive and systematic survey was reported on major crop diseases in Yunnan Province (Duan, 1958).

Prof. Duan was also engaged in the researches of rice bacterial blight and rice blast and found the transmission source of rice bacterial blight for the first time in China. He found that the roots and stems of six kinds of weeds, such as crabgrass, could carry and transmit rice bacterial blight (Pathology Teaching and Research Group of Agronomy Department in Agricultural University, 1976a, b). Later on, this research finding was confirmed by researchers from independent groups in Jiangsu, Zhejiang, and Anhui provinces, attracted the attentions of plant pathologists in China and abroad, and has been written into the textbooks for Universities.

It was still controversial back then on the physiological races of rice blast fungus all over the world. Some researchers in the Philippines asserted that due to the variability of rice blast fungus, it was meaningless to study its physiological races. However, Prof. Duan conducted a detailed study on the variability of rice blast fungus and showed that despite of the variation, rice blast fungus displayed relative stability. He screened out seven rice identification varieties and four strains, and clarified the main physiological races and distribution of rice blast in Yunnan, and also performed pioneer work on the analysis of rice blast resistance genes and the study of genetic laws. In addition, a lot of research has been done on plant viruses (Pathology Teaching and Research Group of Agronomy Department in Agricultural University, 1976; Duan et al, 1979).

Prof. Duan played important roles in organizing international scientific and technological exchanges. In 1981, he invited three famous Japanese pathologists to give lectures in Kunming. 244 students all over China attended these lectures. He personally translated the materials and served as a translator during those lectures. This event was highly praised by the teachers and the students. He also organized 7 large-scale international academic exchanges to strengthen the friendship and scientific connections for plant pathologists from China and Japan.

Prof. Duan has obtained 7 national, provincial and ministerial scientific research awards, and was also elected as the national agricultural labor model and received the special government allowance of the State Council. He also served as an advisor for the Chinese Society of Plant Pathology, chairman of the Southwest Branch of the Chinese Society of Plant Diseases, and chairman of the Yunnan Plant Protection Society. He made outstanding contributions to agricultural education and researches in the Yunnan province.

**Figure 3:**
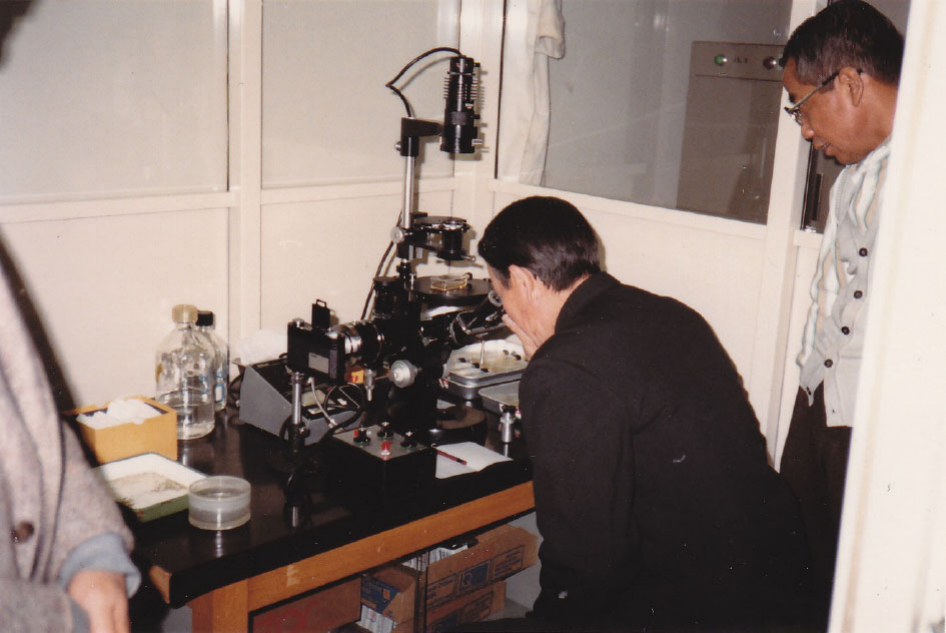
**Prof. Duan was instructing students to use the electron microscope**

Prof. Duan also paid great attention to discipline construction. Since the establishment of the discipline plant pathology at Yunnan Agricultural University, Prof. Duan was committed to laboratory equipment procurement, supervision of researches, and the improvement of teaching and scientific research levels. During the Cultural Revolution, the scientific research conditions were extremely rudimentary. Prof. Duan carried out scientific researches in a simple greenhouse with his colleagues. He also went to the countryside to conduct plant disease investigations, and performed researches on broad bean stem blight in the place borrowed from Agricultural Research Institute and Anning Agricultural Technology Station. Prof. Duan even went to Yuanjiang, where the temperature in summer could reach 40 degrees, and Xishuangbanna, to carry out his studies. The electron microscope has been the dreaming equipment for many teachers and students in the field of plant diseases. At the age of 80, Prof. Duan finally got the approval for the procurement of perspective electron microscope (Fig. 3). The teachers said that they cherish the electron microscope since it reminded them of Prof. Duan.

**Figure 4:**
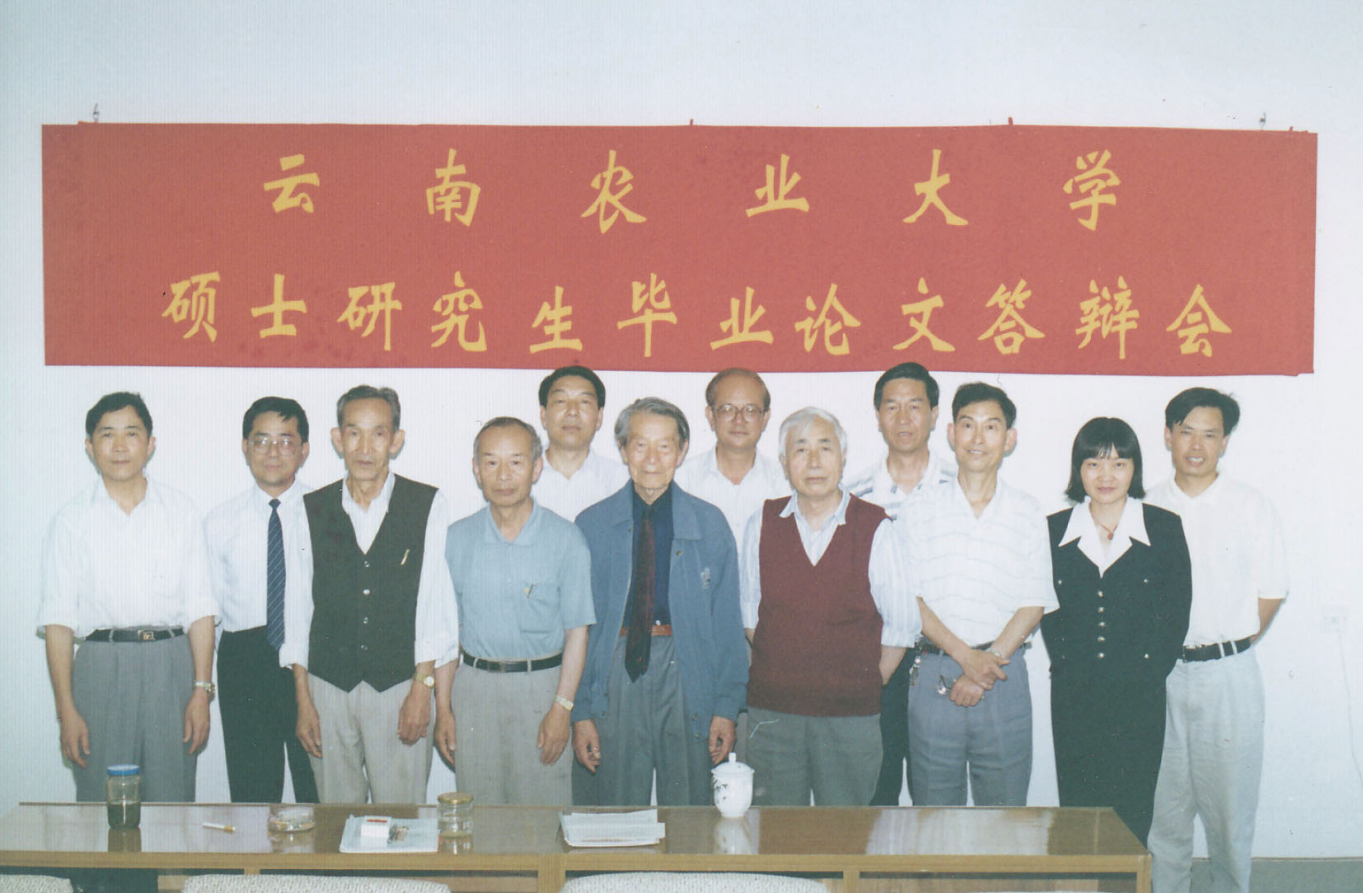
**Prof. Duan (The third from the left in the first row) in his student’s master’s thesis defence**

**Figure 5:**
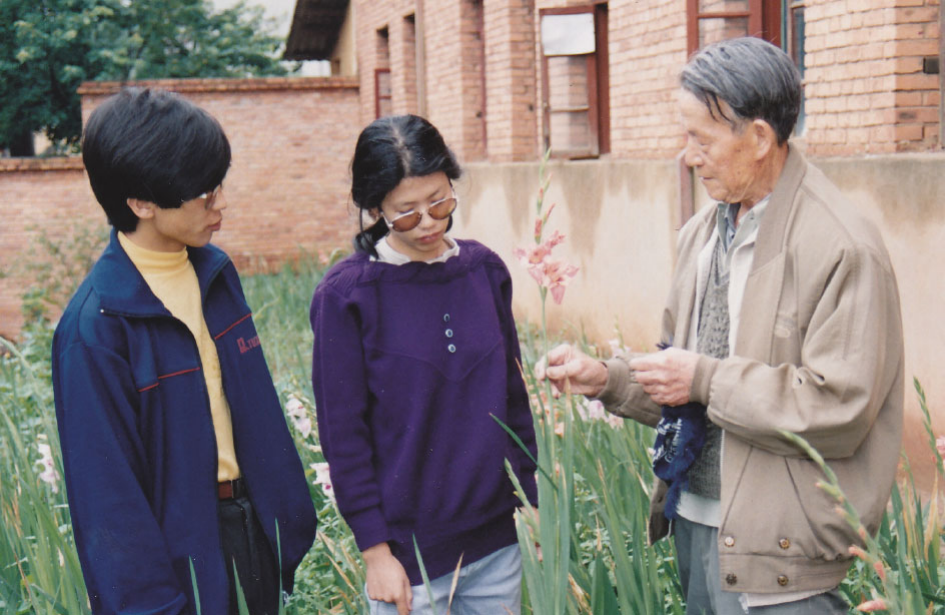
**Prof. Duan was mentoring his students**

Prof. Duan was always concerned about the construction of academic team and made tremendous efforts to strengthen the academic team in plant pathology at Yunnan Agricultural University. He was also a very kind person and was always willing to share his knowledge and experience with the young talents. Under his influence, the department of plant disease has become a united, progressive and ambitious group. This tradition lasted for generations. Mr. Youyong Zhu (朱有勇), the first student of Prof. Duan graduated with a master’s degree, was deeply influenced by him and made great contributions to the pioneer work on the theory of biodiversity control of plant diseases in the academic community, which benefited more than 100 million acres of farmland in Yunnan, Guizhou, Sichuan and other provinces (Fig. 4). Prof. Duan paid so much attention for Mr. Youyong Zhu from undergraduate to master’s degree, from teaching assistant to professor and from key room laboratory director to principal and then to Academician of Chinese Academy of Engineering. Prof. Duan had his own criteria in training graduate students. Firstly, he emphasized the ideological education and required his graduate students to have both scientific ability and political integrity. Secondly, he paid attention is “Words and deeds”, to cultivate his students with an enterprising spirit (Fig. 5). Thirdly, he encouraged his students to not only focus on scientific researches but also participate in teaching (Zhu and Xiao 1986). He also encouraged his students to lead and publish high quality research articles. Last but not least is to take the lead and set the quality of the good articles. He has cultivated lots of young talents, such as the academician Youyong Zhu, who also made great contributions to the agricultural research, is only a representative of Prof. Duan’s effective pedagogy. Many other of Prof. Duan’s disciples have also gone on to contribute greatly to agricultural teaching and research.

Prof. Duan had worked hard in the field of plant pathology for 54 years and made outstanding contributions to the development of plant pathology in China. Prof. Duan has already left us, however, he has given us lots of precious spiritual wealth, such as his moral nobility, selfless dedication, rigorous style of scientific research, compassionate and generous tolerance.
